# Crime and the Human Family*An address delivered at a meeting of the Gross Medical Club, Silver Creek.

**Published:** 1918-09

**Authors:** Henry J. Girvin

**Affiliations:** Chief, Buffalo Police Department, Buffalo, N. Y.


### Crime and the Human Family.*

By HENRY J. GIRVIN,

Chief, Buffalo Police Department, Buffalo, N. Y.

Crime may be defined as the breaking of laws promulgated by God approved and amplified by man.

The Bible tells us that Adam and Eve, tempted of the serpent, ate of the forbidden fruit. Today, under similar circumstances, Eve would be charged with petit larceny, and Adam with aiding and abetting the crime, both conditions being punishable by a fine or imprisonment; thus we find that crime had its inception with the advent of the human race.

If we believe in the laws of heredity, "that like begets like," and in that portion of the second commandment which says, "the sins of the father shall be visited upon the children unto the third and fourth generation," it is very evident that certain impressions of the mind are present at birth; in fact, they are evident long before reasoning power is established; we see them before the baby walks, talks or understands. They are evident, of course, in variable degrees in different individuals, but collectively speaking, it may be said that all children are born with such mental instincts as love, hatred, jealousy, dishonesty and lassitude; all these may lie dormant, or become intensified as development takes place.

In a recent publication a writer states, "We are all born with a crooked streak, and it is only a question of its proximity to the surface as to the amount of damage we do society." This may be considered a radical statement, but from 35 years of dealings with humanity in all its forms, I am most firmly convinced that the writer's opinion is well grounded.

Speaking anatomically, could a careful study be made today of Adam and Eve, but very little difference would be found as compared with the 20th century man or woman, except possibly as to size, form or shape.

But, although there may be no perceptible change evident to the naked eye, one organ has constantly undergone a change and that is the brain. It is to that portion of the human anatomy all our energies must be directed if we are to improve the condition of the human race.

The Declaration of Independence says, "All men are born

*An address delivered at a meeting of the Gross Medical Club, Silver Creek.

equal.'' They are as far as liberty is concerned, providing they obey and respect the law, but as to their equality, as far as intellect is concerned, there is as much difference in the majority as there is between pine and ebony, although the latter are both wood. Too often humanity is judged by appearance, physical powers or weight. Without fear of successful contradiction it may be stated that at every turn we meet individuals who have the appearance of perfect health and sound intellect, but a brief conversation will show at once that their brain has never passed its childhood stage. The child who takes a penny is laughingly pronounced ''clever'' by his foolishly fond parents, repeats the trick, but this time he takes pennies and feeling secure in paternal approval, ever increases the amount until a thief is produced. They did not intend to be dishonest, but they planted the seed of a habit which grew on them until the prison doors closed for the protection of society. The same may be said of jealousy, lassitude and the like; from a small beginning, like the seed of the thistle, they grow and wax strong, and unless early uprooted, choke out and destroy all impulses for good, as the thistles choke out and destroy the tender grain.

A well known authority has said, ''Habits are automatic gratifications of mind impulses,'' in other words, the mind directs what the tongue, hands or feet carry out. It will be admitted that but one perfect man has ever lived, consequently we must not expect too much of the present or coming generation, yet we must strive to keep the human race in as nearly a perfect condition as possible, and to use all our energies for its betterment.

This one thing can be said with a reasonable assurance of reality, the first impressions are the ones that last; teaching should commence with the babe in the cradle.

Although there is no doubt in the minds of police officials who have made a study of crimes that the great majority of noted or habitual criminals are born such, education must not stop, for the saving of even one of these from a life of crime increases security in the mind of society and a saving of thousands of dollars to the taxpayers.

We must not lose sight of the fact that we belong to the animal kingdom; that we are only removed from the dumb brute in that we have the power of reason and can articulate. With this unquestionable truth ever before us, it is unreasonable to ask that we should be given the same chance that they are; that is, as to procreation.

After the child is born, discipline is paramount as to habit formation, especially so for those so prone to arise from the inherited instincts of a morbid mind.

It has been written, and President Wilson used practically these words in a recent speech, "To have people good, if it is necessary to do so, make them afraid of you." This may seem a harsh statement; other words might be used for fear, but to one whose duty it is to protect society from those who have formed deleterious habits, there can be no questioning of the necessity of the aforementioned statements.

A police officer's duty can be defined as; first, the protection of life and property; second, the prevention of crime; third, taking into custody people who have committed crime. Criminals fear three things, officers, publicity and the deprivation of freedom. A crime is never committed, unless by accident, in the presence of an officer, in fact, they avoid officers at all times; it is a true saying, "That a guilty conscience is its own accuser." The innocent and law abiding should have nothing to fear; the officer is their best friend, and he should always be considered as such.

May I give you an example of publicity? Since instituting at my headquarters in Buffalo, the screen, where every morning at 9 o'clock all criminals arrested during the previous 24 hours are shown under a strong electric light to our detectives, whom they cannot see, visits to our city by noted criminals have decreased fully 50 per cent, in fact, those who have never appeared on the screen would prefer 30 days in the penitentiary to another appearance there.

We all realize what deprivation of freedom means, especially when this may be for years, or for ones natural life. Many thousand men and women are detained in the penal institutions of this country, yet it can scarcely be said that crime is on the increase when we take into consideration the increase in population, and that every civilized country on the globe has representatives here.

As to treatment; many physicians advocate, and many operative measures have been taken for fractures of the skull, which are supposed to be the cause of a criminal career, but to my knowledge so far, they have been a failure. As yet no scientist has brought forward a serum; I know of no medicine for the purpose, may I therefore sum up the foregoing statements as follows:

In the procreation of the race we should be given the same chance as is our four-footed friends, especially of the higher order; mothers and fathers should look more closely into the life history of prospective son and daughter-in-laws; they should not stop at the immediate family, but as the late Henry Ward Beecher once said, "Go back to the grandparents."

Humanity is guided through these tortuous paths of life; first, by inherited instinct; second, the sphere in which we live. The first we find controlled by inherited conditions of the mind; the previously transmitted instincts which vary in form from trivial wrong-doing to the crime of murder; they are due to habit formation.

Second, the sphere in which we live, or environments; this is the most fertile field of habit formation.

It therefore behooves us as fathers, mothers and later, as teachers to use our very best endeavors to discipline the child against the formation of deleterious habits which are the ground work of practically all the crime committed today; such discipline should begin at the cradle.

I wish to take the liberty at this time to thank several members of the medical profession for the valuable aid given me in the sale of Liberty Bonds, War Savings Stamps, and protection of the public; Capt. Jacob Goldberg, Lieut. J. Henry Dowd, Sergeants Dewitt H. Sherman and J. F. Rice.

Headquarters of Police, Buffalo, N. Y.

---

**New Poison Gas.** Teulieres, Jour. de Med. de Bordeaux, Feb., calls this gas "sulfure d'ethyl bichlore," which we are unable to translate into chemical terms. Among 1500 men exposed, a third presented insignificant lesions and only 23 cases were serious, involving the eyes. In most cases, the phtophobia subsided in 4 or 5 days, cure being complete in 2-3 weeks, the lesion consisting merely of a rather severe conjunctivitis. In all, there were 3 ulcerations of the cornea and 1 panophthalmitis.

---

**Chyluria.** Loumeau, Jour. de Med. de Bordeaux, Feb., reports the case of a man 60 who had had chyluria since a gonorrhoea at the age of 27. Cystotomy revealed the presence of an intra-prostatic adenoma which was enucleated. Death followed and a partial necropsy revealed neither prevertebral adenopathies nor lymphangectases. A single previous examination had shown no filariae but, in view of the fact that the patient was a native of Mauritia, the chyluria was considered as probably of this nature. Renal symphysis with a single ureter was found to exist.

---

**Intestinal Occlusion With Biliary Calculus.** Villar, Jour. de Med. de Bordeaux, Feb., operated for intestinal obstruction 8 days after an attack of colic. A mass had been found behind the uterus, by rectal examination. A biliary calculus, the size of a hen's egg, weighing 95 grams, was found in the small intestine. Death ensued the next day. Begouin reported an identical case.

